# Regulatory network of inflammation downstream of proteinase-activated receptors

**DOI:** 10.1186/1472-6793-7-3

**Published:** 2007-03-30

**Authors:** Ricardo Saban, Michael R D'Andrea, Patricia Andrade-Gordon, Claudia K Derian, Igor Dozmorov, Michael A Ihnat, Robert E Hurst, Cindy Simpson, Marcia R Saban

**Affiliations:** 1 Department of Physiology, The University Oklahoma Health Sciences Center, Oklahoma City, OK 73104, USA; 2 J&J Pharmaceutical Research and Development Spring House, PA 19477-0776, USA; 3 Oklahoma Medical Research Foundation (OMRF), Arthritis and Immunology Research Program, Microarray/Euk. Genomics Core Facility, Oklahoma City, Oklahoma 73104, USA; 4 Department of Cell Biology, The University Oklahoma Health Sciences Center, Oklahoma City, OK 73104, USA; 5 Department of Urology, The University Oklahoma Health Sciences Center, Oklahoma City, OK 73104, USA

## Abstract

**Background:**

Protease-activated receptors (PAR) are present in the urinary bladder, and their expression is altered in response to inflammation. PARs are a unique class of G protein-coupled that carry their own ligands, which remain cryptic until unmasked by proteolytic cleavage. Although the canonical signal transduction pathway downstream of PAR activation and coupling with various G proteins is known and leads to the rapid transcription of genes involved in inflammation, the effect of PAR activation on the downstream transcriptome is unknown.

We have shown that intravesical administration of PAR-activating peptides leads to an inflammatory reaction characterized by edema and granulocyte infiltration. Moreover, the inflammatory response to intravesical instillation of known pro-inflammatory stimuli such as *E. coli *lipopolysaccharide (LPS), substance P (SP), and antigen was strongly attenuated by PAR1- and to a lesser extent by PAR2-deficiency.

**Results:**

Here, cDNA array experiments determined inflammatory genes whose expression is dependent on PAR1 activation. For this purpose, we compared the alteration in gene expression in wild type and PAR1^-/- ^mice induced by classical pro-inflammatory stimuli (LPS, SP, and antigen). 75 transcripts were considered to be dependent on PAR-1 activation and further annotated *in silico *by Ingenuity Pathways Analysis (IPA) and gene ontology (GO). Selected transcripts were target validated by quantitative PCR (Q-PCR). Among PAR1-dependent transcripts, the following have been implicated in the inflammatory process: *b2m, ccl7, cd200, cd63, cdbpd, cfl1, dusp1, fkbp1a, fth1, hspb1, marcksl1, mmp2, myo5a, nfkbia, pax1, plaur, ppia, ptpn1, ptprcap, s100a10, sim2*, and *tnfaip2*. However, a balanced response to signals of injury requires a transient cellular activation of a panel of genes together with inhibitory systems that temper the overwhelming inflammation. In this context, the activation of genes such as *dusp1 *and *nfkbia *seems to counter-balance the inflammatory response to PAR activation by limiting prolonged activation of p38 MAPK and increased cytokine production. In contrast, transcripts such as *arf6 and dcnt1 *that are involved in the mechanism of PAR re-sensitization would tend to perpetuate the inflammatory reaction in response to common pro-inflammatory stimuli.

**Conclusion:**

The combination of cDNA array results and genomic networks reveals an overriding participation of PAR1 in bladder inflammation, provides a working model for the involvement of downstream signaling, and evokes testable hypotheses regarding the transcriptome downstream of PAR1 activation.

It remains to be determined whether or not mechanisms targeting PAR1 gene silencing or PAR1 blockade will ameliorate the clinical manifestation of cystitis.

## Background

In general, inflammation plays a role in most bladder pathologies, including bladder cancer [1-4], and represents a defensive reaction to injury caused by physical damage, chemical substances, micro-organisms, or other agents [1,2]. In particular, neurogenic bladder inflammation involves the participation of mast cells and sensory nerves. We previously presented evidence indicating a key role for mast cells and their products in bladder inflammation [5-7]. As a consequence of inflammation, products of mast cell degranulation such as tryptase can be found in the urine of both bladder cancer and cystitis patients [8]. In addition to tryptase, other serine proteases such as thrombin and trypsin are produced during tissue damage and make important contributions to tissue responses to injury, repair, cell survival, inflammation [9-12], and pain [13-17]. Tissue responses to these enzymes are modulated by protease-activated receptors (PARs), a unique class of G protein-coupled receptors that use a fascinating mechanism to convert an extracellular proteolytic cleavage event into a trans-membrane signal. These receptors carry their own ligands, which remain cryptic until unmasked by receptor cleavage (for a review, please see references [13,16,18,19]).

In order to better understand the role of PARs in cystitis, we used a well established mouse model [20-22] to determine the relative effect of PAR-specific peptide agonists. Comparison of inflammatory responses in wild type, PAR1- and PAR2-deficient mice, revealed a mandatory role of PAR1 and, to a lesser extent, PAR2 in mediating bladder responses to a variety of pro-inflammatory stimuli [23].

Four PARs have been cloned to date, and all four PARs are co-expressed in the mouse bladder urothelium [24], with PAR2 and PAR3 being the most abundant in the bladder epithelial layer. In addition to the urothelium, PAR1 and PAR2 are also expressed in mouse detrusor muscle, and PAR4 is expressed in mouse peripheral nerves and plexus cell bodies [24]. Similarly, in rats PAR2, 3, and 4 are expressed in urothelium, detrusor muscle, and bladder nerve fibers, and bladder afferent cells in dorsal root ganglia express PAR2 to 4 [25]. Confocal microscopy has revealed the co-localization of PAR2, 3, and 4 with protein gene products 9.5 and vanilloid receptor 1, suggesting that PARs are distributed in C-fiber bladder nerves [25].

In addition, PARs are differentially modulated during mouse bladder inflammation. Urothelial PAR2 and, to a lesser extent, PAR1 are down-regulated in acute inflammation, whereas PAR3 and PAR4 are up-regulated [24]. Bladder fibroblasts were found to present a clear demarcation in PAR expression in response to acute and chronic inflammation [24]. Additional evidence for the participation of PARs in the bladder inflammatory response was the finding that known pro-inflammatory stimuli such as LPS, substance P, and antigen challenge induce an increase in PAR4 RNA within four hours [20]. Upregulation of PAR protein levels has been shown to be part of rat bladder responses to cyclophosphamide [25]. Since PAR1 is well represented in the urinary bladder [24] and its expression is altered in bladder inflammation [24], we set forth to determine the molecular pathways downstream of PAR1 activation. For this purpose, we used a combination of gene-array technology, data mining using Ingenuity Pathways Analysis (IPA), and gene ontology (GO) annotation.

The signal transduction pathway downstream of PAR activation and coupling with various G proteins is known and leads to the rapid transcription of genes involved in inflammation [13,26]. However, the exact composition of the transcriptome downstream of PAR activation remains to be determined. Our approach revealed a cascade of PAR1-dependent transcripts involved in apoptosis, cell death, cell cycle, cell growth and proliferation, cell motility, cell-cell interaction, gene expression, immune response, inflammation, renal and urologic development and disease, hematological disease, and cancer.

The combination of cDNA arrays and *in silico *genomics network analysis reveals an overriding participation of PAR1 receptors in bladder inflammation, provides a working model for the involvement of downstream transcripts, and evokes testable hypotheses regarding the transcriptome downstream of PAR1 activation.

## Results

### Genes downstream of PAR1 activation

**Figure 1:**
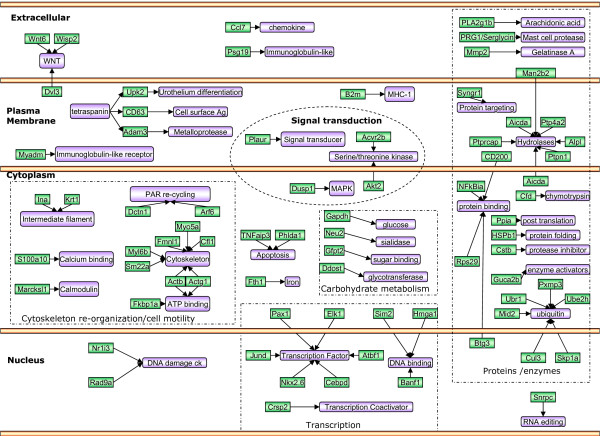
**A. PAR1-dependent genes**. Inflammation was induced by intravesical instillation of substance P or LPS into the bladder of WT and PAR1^-/- ^mice and compared to control (saline-treated); see methods for details. Twenty-four hours after, bladders were removed and the RNA was isolated for cDNA array experiments. Genes that were upregulated in response to inflammation in wild type mice and failed to be up-regulated in PAR1^-/- ^mice were considered to be PAR-1 dependent transcripts. PAR-1 dependent transcripts were used to query the Ingenuity Pathways Analysis [IPA] and based on EntrezGene and Gene Ontology annotations were distributed into relevant biological compartments and biological interactions.

**Figure 2:**
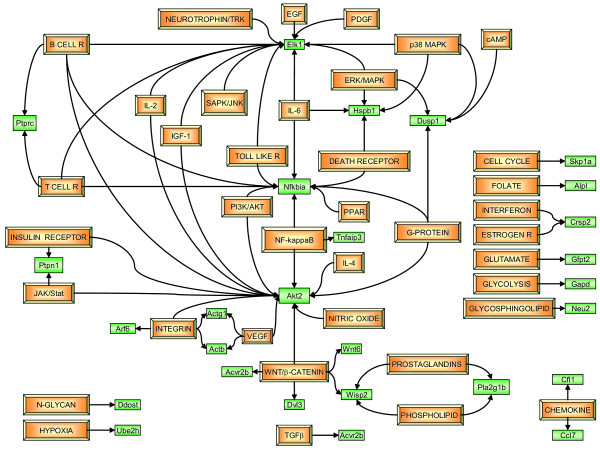
**Participation of PAR1-dependent genes in canonical pathways**. The experimental datasets of PAR1-dependent genes was used to query the Ingenuity Pathways Analysis [IPA] and overlaid to canonical pathways.

**Figure 3:**
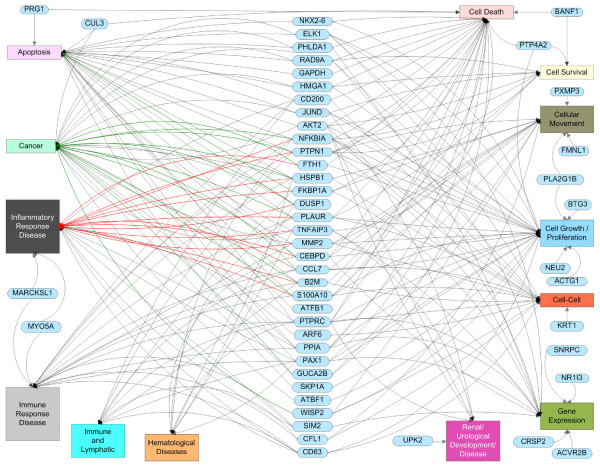
**Primary functions associated with PAR1-dependent genes**. The experimental datasets of PAR1-dependent genes was used to query the Ingenuity Pathways Analysis [IPA] and overlaid with primary functions and diseases.

**Figure 4:**
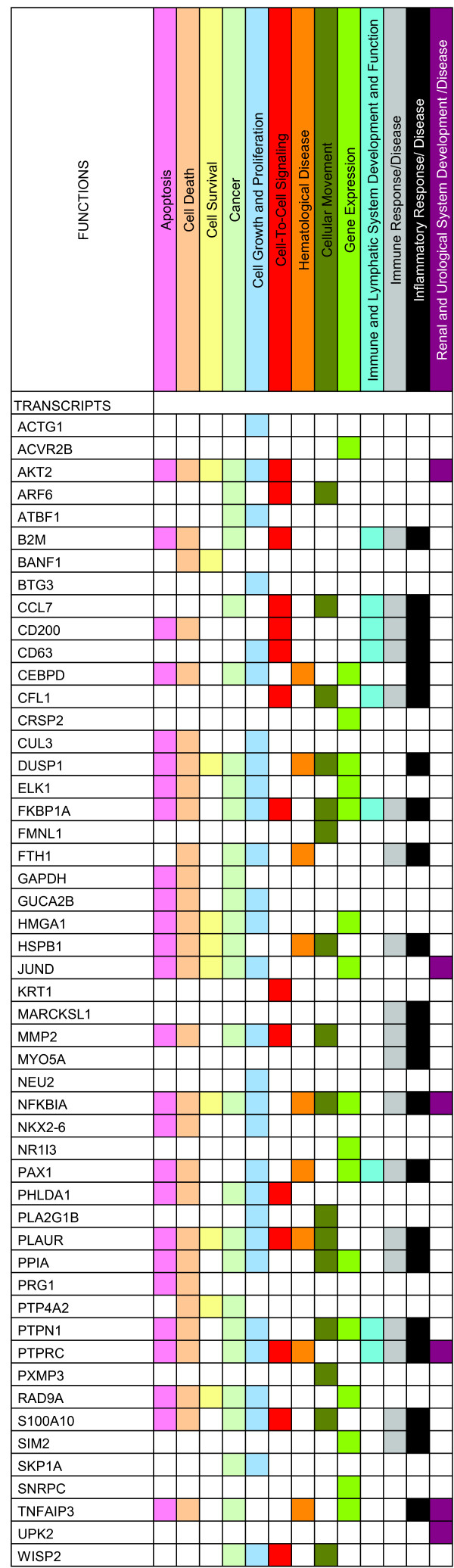
Functions and disease shared by PAR1-depend transcripts.

Seventy five genes fulfilled both the criteria of being expressed 3-fold higher after stimulation with SP or LPS and of not changing expression in response to the same stimuli of PAR1^-/- ^mice, and were considered to be PAR1-dependent (Additional files 1 and 2, Table 1A and 1B). To further annotate this set of genes, we used a web-based entry tool developed by Ingenuity Pathways Analysis [IPA] [27]to query their knowledge database [27-29]. The resulting networks contain: **A**- Sub-cellular layout indicating the predominant location for the expression of encoded proteins (Figure 1); **B**-Analysis of canonical pathways significantly associated with this group of genes (Figure 2), and **C-D- **Biological functions across the entire dataset most significantly associated with this set of genes (Figure 3 and 4).

### Sub cellular location (Figure 1)

Genes (green) were associated with function (magenta) and localized to each compartment according to IPA and gene ontology. Dotted lines further group the genes by activity or function such as signal transduction, cytoskeleton re-organization and cell motility, carbohydrate metabolism, proteins and enzymes, transcription, and RNA editing. Of note, a particular set of genes such as *dctn1 *and *arf6 *are involved in PAR trafficking, and their function is associated with other PAR1-dependent transcripts with function in the cytoskeleton reorganization.

### Participation of PAR1-dependent genes in canonical pathways (Figure 2)

Overall, PAR1-dependent transcripts belong to several canonical pathways (Figure 2). This type of IPA also revealed key genes that are significantly associated with more than one canonical pathway. This is the case of *elk1, nfkbia, hspb1, dusp1, and akt2*. In addition, this analysis indicates pathways such as VEGF and integrins which share common genes, as is the case of those encoding cytoskeleton proteins *actg1 *and *actb*.

### Primary functions associated with PAR1-dependent genes (Figure 3 and 4)

Figures 3 and 4 represent biological functions significantly (p < 0.01) associated with PAR1-specific genes. Those included: apoptosis (n = 26 genes); cell death (n = 29); cell survival (n = 10); cancer (n = 29); cellular growth and proliferation (n = 29); cell-to-cell signaling (n = 15); hematological disease (n = 9); cellular movement (n = 16); gene expression (n = 18); immune and lymphatic system development and function (n = 9); immune response/disease (n = 19); and inflammation and inflammatory disease (n = 22). This type of analysis also revealed PAR-1 dependent genes (*upk2, jundD, ptprc*, and *nfkbp1a) *encoding proteins associated with renal and urological diseases. Figure 1D also indicates common pathways of PAR1-dependent transcripts shared by inflammation (red) and cancer (green) and revealed possible targets uniquely associated with some of the biological functions.

### Target validation by Q-PCR of Chromatin Immunoprecipitation (CHIP)-based assays

**Figure 5:**
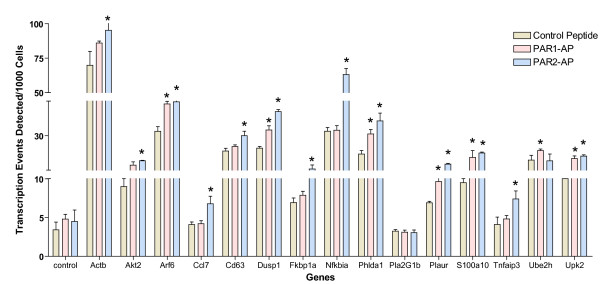
**Target validation by Q-PCR of Chromatin Immunoprecipitation (CHIP)-Based Assays**. For target validation, female C57BL/6J mice (n = 20 per group) were instilled with 200 μl of one of the following substances: control inactive peptide (LRGILS [94]) or PAR-activating peptides (PAR1-AP = SFFLRN [94]; PAR2-AP = SLIGRL [94]). Twenty-four hours after instillation, bladders were removed and frozen. Bladders were exposed briefly to formaldehyde for cross-linking of the proteins and DNA together, followed by sonication to fragment the DNA. An antibody against RNA polymerase II (Abcam) was then used to precipitate the DNA transcriptome that was isolated and then purified using phenol extraction and EtOH precipitation. The final CHIP DNAs were then used as templates for Q-PCR reactions using primer pairs specific for each gene of interest, additional material 2 (Table 2). Q-PCRs were run in triplicate and the averaged Ct values were transferred into copy numbers of DNA using a standard curve of genomic DNA with known copy numbers. The resulting transcription values for each gene were also normalized for primer pair amplification efficiency using the Q-PCR values obtained with Input DNA (un-precipitated genomic DNA). Results are presented as "transcription events detected per 1000 cells" for each gene tested. Error bars correspond to standard deviations from the triplicate Q-PCR reactions. Control represents an un-transcribed region of the genome. Asterisks indicate a statistical significant difference (p < 0.05).

Of the 19 genes tested by Q-PCR analysis of CHIP isolated from wild type mice challenged with control peptide, PAR1- and PAR2-AP, 4 genes (*adam-3, dctn1, elk1*, and *mmp2*) had their control levels 1.5 times below background (un-transcribed region) and, therefore, their results are not being presented. Results are presented in figure 5 as averaged Transcription Events Detected Per 1000 Cells for each gene tested and their standard deviations. With the exception of *pla2G1b*, these results indicate that treatment of wild type mice with PAR1- and PAR2-AP induced up-regulation of the following PAR1-dependent genes: *actb, akt2, arf6, ccl7, cd63, dusp1, fkbp1a, nfkbia, phlda1, plaur, s100a10, tnfaip3, ube2h*, and *upk2*.

### PAR-activation of calcium independent PLA_2 _(figures 6 and 7)

**Figure 6:**
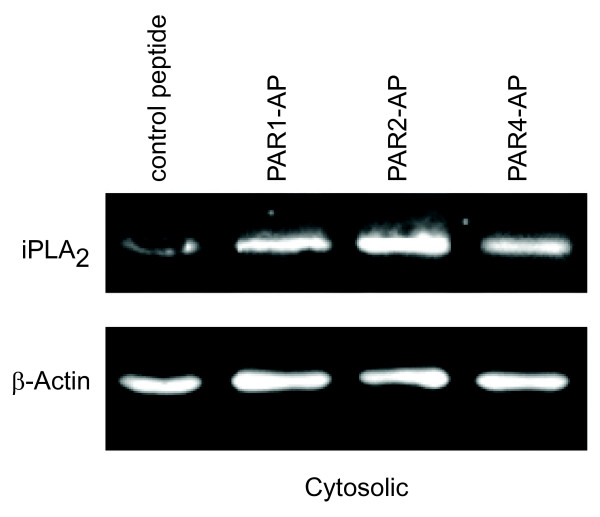
**PAR-APs induce up-regulation of iPLA_2 _in the mouse urinary bladder**. Cytoplasmic extract from the bladder mucosa of wild type mice instilled with PAR-APs- or control peptide were subjected to Western Blot analysis to measure relative concentration of iPLA_2_. Results are representative of four separate experiments.

**Figure 7:**
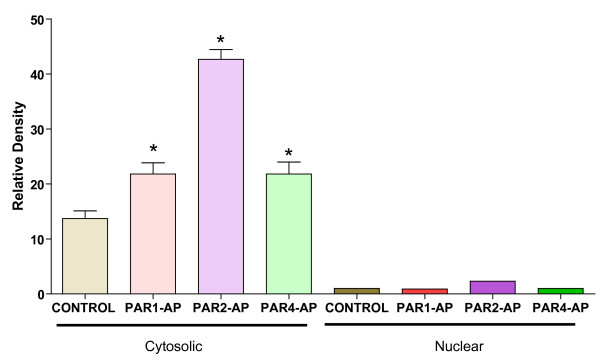
Average densitometric values of PAR-APs induce up-regulation of iPLA_2 _in the mouse urinary bladder (Figure 6).

Our cDNA array results indicate that *pla2G1b *encoding a calcium-dependent PLA_2 _was a PAR1-dependent transcript. However, this particular transcript was not validated by Q-PCR of actively-transcribed DNA (Figure 5). Because of the prominent role of the calcium-independent phospholipase A_2 _(iPLA_2_) in mediating tryptase-activated PAR in bladder microvascular endothelial cells [30], bladder urothelial cells [31,32], and PAR2-AP in bladder contractions [33], we decided to determine whether bladder instillation with PAR-APs also induced up-regulation of iPLA_2 _in the mouse bladder mucosa. The rationale for using only the mucosa was to decrease the complexity of the bladder system, getting closer to the urothelial cell in culture used by the other authors [31,32]. For this purpose, cytoplasmic extracts from the bladder mucosa of WT mice instilled with PAR-APs- or control peptide- were subjected to Western Blot analysis to measure relative concentration of iPLA_2_. Results are representative of 4 separate experiments (Figure 6). The bar graph shows the average densitometric values (Figure 7). When data was normalized with beta-actin used as loading control, the same relationship was observed (data not shown). These data indicate that in the mouse urinary bladder, all PAR-APs induced up-regulation of iPLA_2_.

## Discussion

Serine proteases that originate from the circulation (coagulation factors), inflammatory cells (mast cell tryptase, neutrophil granzyme A, and proteinase 3), and epithelial and neuronal tissues (trypsins) can specifically regulate cells by cleaving PARs [34]. Proteases cleave PARs to reveal tethered ligand domains that bind to and activate the cleaved receptors. The proteases that activate PARs are often generated and secreted during injury and inflammation, and PARs orchestrate tissue responses to these insults, including hemostasis, inflammation, nociception, and repair mechanisms [34].

The present results indicated fundamental alterations elicited by pro-inflammatory substances (LPS and SP) in bladder gene-regulation that are mediated by activation of PAR1. The possible mechanisms by which LPS and SP induce PAR activation include degranulation of mast cells with a concomitant release of tryptase and plasma extravasation leading to accumulation of products of the coagulation cascade that activate PARs. However, the activation of PAR in the physiological state is more complex. Although thrombin is a recognized physiological activator of PAR1 and PAR4, the endogenous enzymes responsible for activating PAR2 in urinary bladder are not known. Recently, it was demonstrated that tissue kallikrein family of proteinases are able to regulate PAR signaling and may represent important endogenous regulators PAR1, PAR2, and PAR4 [35]. Interestingly, the kallikrein family plays a fundamental role in bladder physiology [36].

### Transcriptional alteration downstream PAR activation

In terms of mechanism of action, it is well established that after stimulation, PARs couple to various G proteins and activate signal transduction pathways resulting in the rapid transcription of genes that are involved in inflammation [13,26,34,37]. However, the response of the transcriptome downstream of PAR activation is not known. Here, we used an approach involving the combination of cDNA arrays, CHIP assay, and *in silico *querying of knowledge databases in order to identify functions dependent of PAR1 activation that are modified during bladder inflammation. Although we clearly defined the criterion for a gene to be named PAR1-dependent (see Methods section), the expression of some/many of these genes may only be indirectly dependent on PAR-1, due to the nature of the downstream cascades and interacting pathways.

The use of cDNA arrays has contributed immensely to the understanding of inflammation, in general, and the bladder inflammatory transcriptome, in particular [7,20,21,38-41]. In addition, the use of curated networks such as the Ingenuity Pathways Analysis leads to a comprehensive integration of how PAR1-dependent transcripts correlate with canonical pathways and known biological functions. Because our results were obtained with whole bladders, it is not clear whether any single network may be operative in a particular cell type. However, this approach can potentially identify previously unrecognized connections among pathways and, therefore, suggests new hypotheses for the mechanisms of bladder inflammation. Therefore, the value of our approach is to raise testable hypotheses that can be tested in isolated cells or in individual bladder layers. Aberrant expression of protease-activated receptors (PARs) has been associated not only with inflammation but with increased angiogenesis, tumor growth, and metastasis of various cancers [42-47]. Therefore, our approach outlined a global visualization of PAR-dependent transcripts (Figure 1) as well as their interaction with several pathways (Figures 2, 3, 4).

Our results indicated that several genes known to be part of the inflammatory response were found downstream of PAR1 activation (*b2m, ccl7, cd200, cd63, cdbpd, cfl1, dusp1, fkbp1a, fth1, hspb1, marcksl1, mmp2, myo5a, nfkbia, pax1, plaur, ppia, ptpn1, ptprcap, s100a10, sim2*, and *tnfaip2)*. The present work adds to this list pro-inflammatory genes such as *wnt6, wisp2, and dvl3 *that belong to the *WNT *family known to be altered in human interstitial cystitis [48,49]. In contrast, some of the proteins encoded by PAR1-dependent transcripts play a role in shutting down the inflammatory cascade. This is the case with type-1 membrane glycoprotein encoded by *Cd200*, which delivers an inhibitory signal cancelling the inflammation-induced macrophage activation [50]. Within this group, we also highlight *nfkbia*, which encodes IkappaB-alpha, an inhibitor of the NF-kappaB cascade [51,52].

In addition to genes involved in inflammation, we found upregulation of members of the *tetraspanin *family (*cd63*, and *adam3*) and upk2 that dimerises with the tetraspanin uroplakin 1a. *Cd63 *encodes a protein localized in the membrane of mast cells [53], and anti-CD63 antibodies inhibit mast cell adhesion to fibronectin and vitronectin [53]. Adam-3 may have a role in inflammation by regulating the expression of allograft inflammatory factor-1 and iNOS [54]. *Uroplakin 2 *is a major component of the surface plaques of the urothelium [55], is found exclusively in differentiated mammalian urothelium [56], and is a product of terminally-differentiated apical cell layer [57,58]. In addition to differentiation, uroplakins play a fundamental role in the bladder permeability barrier [59], wound healing [60], bacterial adherence and infection [61], and possibility bladder inflammation [62]. The present results are the first direct evidence indicating that inflammation *per se *alters the message for uroplakin 2.

Among the secreted enzymes regulated by PAR1-dependent transcripts is the calcium-dependent phospholipase A_2_, group IB that was up-regulated by LPS- and SP-induced bladder inflammation in wild type mice. Our Q-PCR results did not confirm an up-regulation of transcription in response to PAR1-AP and PAR2-AP. There are several explanations for this discrepancy. One possibility is that the differential expression of RNA was not due to active transcription and therefore, reflects RNA processing. As others have shown that the calcium independent phospholipase A_2 _(iPLA_2_) mediates bladder urothelial [31], microvascular [30], and smooth muscle [33] responses to PAR activation, and has a role in promoting mmp-2-induced cell migration via the phosphatidylinositol 3-kinase – Akt pathway [63], we went further to test whether this calcium-independent PLA is also up-regulated in response to PAR-APs. Our results confirmed upregulation of iPLA_2 _protein in the bladder mucosa of mice instilled with PAR-APs, supporting the upregulation of message levels.

Another group of genes encodes proteins with roles in signal transduction pathways. Those include: *plaur, actR-IIb, dusp1*, and *akt2. Plaur *encodes the receptor for urokinase plasminogen activator *(u-PA receptor) *and promotes plasmin formation [64]. *Plaur *is most closely associated with cancer invasion [65]. A strong correlation exists between PAR mediating the response of thrombin and u-PAR-mediated cancer cell migration/invasion [42]. *Dusp1 *encodes a dual specificity phosphatase 1 or mitogen-activated protein kinase [MAPK] phosphatase 1, which dephosphorylates and inactivates MAPKs [66,67].*Dusp1 *regulates a subset of LPS-induced genes [68] and modulates the anti-inflammatory effects of dexamethasone in macrophages [66]. *Akt2 *is a putative oncogene encoding a protein belonging to a subfamily of serine/threonine kinases containing SH2-like (Src homology 2-like) domains. Others have shown that thrombin and PAR- APs induce *Akt2 *phosphorylation [69,70]. *Akt2 *is a general protein kinase capable of phosphorylating several known proteins and, therefore, occupies a central position in several signal transduction pathways (Figures 1B and 1C). Moreover, PAR activation in platelets leads to Akt2 phosphorylation by a mechanism involving either G(12/13) [69] or G_i _[71] and Src kinase activation [69].

The process of desensitization and re-sensitization of PA receptors is being elucidated [72]. Agonists of PARs induce an irreversible activation by proteolytic cleavage of the tethered ligand peptides and rapid receptor endocytosis that are targeted to lysosomes [73,74]. Interestingly, among the PAR1-dependent transcripts, the following encode proteins of the ubiquitin pathway: *mid2, ubr1, pxmp3, ube2h, cul3, and skp1a*. Little is known regarding the alteration of expression of this set of genes during inflammation. However, it has been reported that TNFα stimulates *ube2h *expression by a mechanism involving NF-kappaB [75]. Together these results raised the intriguing hypothesis that inflammation can induce an increase in the ubiquitin pathway that also modulates the fate of PARs [76].

*The Rab *family of proteins play a fundamental role in PAR re-sensitization [77]. Here, we show that *dcnt1 *and *arf6 *also involved in PAR trafficking and are increased during inflammation. *Dcnt1 *is a subunit of the dynein/dynactin complex and an effector for *rab6 *[78]. This protein is required for the retrograde movement of vesicles and organelles along microtubules [79]. *Arf6 *is a member of the human ADP-ribosylation factor (*ARF*) family of proteins belonging to the Ras superfamily of small GTPase implicated in vesicle trafficking [80-82]. *Arf6 *participates in the endosomal pathway that regulates endocytosis of several receptors [83]. Post-internalization, *arf6 *and other membrane components are recycled back to the cell surface [80]. In addition to membrane trafficking, *arf6 *cellular functions include: actin remodeling, cell adhesion, redistribution of β1 integrins, phagocytosis, cell division, and tumor-cell invasion (for a review, see [80]).

As alterations in PAR density itself [25] or in the mechanisms involved in re-sensitization of these receptors can have strong consequences in the shift between homeostasis and inflammation, our results raise the intriguing hypothesis that, in contrast to PAR endocytosis and cessation of the stimulus that occurs during normal physiological responses, inflammation may lead to increased re-cycling of PARs back to the plasma membrane and, therefore, perpetuation of the signal transduction downstream of PAR activation.

Although the classification by biological function permits a visual association of genes and cellular processes (Figure 1), it has the disadvantage of over simplification because several genes may belong to more than one group. This is the case of *mmp2 *(gelatinase A) that is involved in the breakdown of extracellular matrix in normal physiological processes, such as embryonic development, reproduction, and tissue remodeling, as well as in disease processes such as inflammation and cancer. Therefore, we constructed figures 2, 3, and 4 in order to illustrate common pathways involving PAR1-dependent transcripts. This type of association analyses permits the identification of new pathways such as the interaction of PAR1-dependent transcripts with the VEGF-family of growth factors, and suggests explanations of an active role of PAR in angiogenesis [45,84-86]. Together, the pathway analysis revealed relevant target for therapeutic interventions to control or to prevent disease progression and bladder inflammation.

### Other cDNA array analysis

We are aware of a single publication investigating genes downstream of PAR1 activation. This work was performed in a human endothelial cell line challenged with thrombin with the aim of identifying early genes and comparing to those up-regulated in response to leukotriene D4 [87]. Unfortunately, the referred work did not provide GenBank accession numbers for the PAR1 activated transcripts, which makes comparisons of the results to the current study difficult. Nevertheless, some similarities could be found between their work and the present one. This is the case of *dusp1 *that was found upregulated in both manuscripts. In addition we found adam3, whereas Uzonyi, et al. found another disintegrin-like metalloprotease (adamts1) [87].

## Conclusion

This work indicates an overriding participation of PAR1 receptors in bladder inflammation, provides a working model for the involvement of a network of transcripts downstream of PAR1 activation, and evokes testable hypotheses regarding the regulation of PAR. In this context, the activation of genes such as *dusp1 *and *nfkbia *seems to counter balance the inflammatory response to PAR activation by avoiding prolonged activation of p38 MAPK and increased cytokine production [68]. In contrast, transcripts such as *arf6 and dcnt1 *that are involved in the mechanism of receptor re-sensitization would tend to perpetuate the inflammatory reaction. It remains to be determined whether PAR1 receptor blockade or selective gene silencing transcripts downstream of PAR activation will ameliorate the clinical manifestation of cystitis. Inhibiting of PAR up-regulation using small interfering RNA technology, as confirmed by immunoblotting, should substantially reduced bladder inflammatory response as it has been shown in other systems [88].

## Methods

### Animals

All animal experimentation described here was performed in conformity with the "Guiding Principles for Research Involving Animals and Human Beings" (OUHSC Animal Care & Use Committee protocol #05-088I). PAR1^-/-^[89] and C57BL/6J mice were used in this research. C57BL/6J mice were used as wild type since PAR1^-/- ^was enriched in this background.

### Induction of cystitis

Acute cystitis was induced in groups of mice, as we described previously [20-22,24,40]. Briefly, female wild type (C57BL/6J), and PAR-1^-/- ^mice were anesthetized (ketamine 200 mg/kg and xylazine 2.5 mg/kg, i.p.), then transurethrally catheterized (24 Ga.; 3/4 in; Angiocath, Becton Dickson, Sandy, Utah), and the urine was drained by applying slight digital pressure to the lower abdomen. The urinary bladders were instilled with 200 μl of one of the following substances: pyrogen-free saline, SP (10 μM), or *Escherichia coli *LPS strain 055:B5 (Sigma, St. Louis, MO; 100 μg/ml). Substances were infused at a slow rate to avoid trauma and vesicoureteral reflux (18). To ensure consistent contact of substances with the bladder, infusion was repeated twice within a 30-min interval, and a 1-ml tuberculin syringe was maintained on the catheter to retain the intravesical solution for 1 hour. After that the catheter was removed and mice were allowed to void normally. Twenty-four hours after instillation, mice were euthanized with pentobarbital (200 mg/kg, i.p.) and bladders removed rapidly.

### Minimum information about microarray experiments – MIAME [90]

#### Objective

To determine the time course of gene-expression in urinary bladders in response to saline, SP, or LPS, challenge of wild type control and PAR1^-/- ^mice.

#### Array design

Mouse plastic 5 K Arrays (Clontech, Palo Alto, CA, Cat. #634809). For a complete list of genes present in this array see reference [91].

#### Animal numbers

Female WT and PAR1^-/- ^mice were instilled with saline, SP (10 μM), or *Escherichia coli *LPS strain 055:B5 (Sigma, St. Louis, MO; 100 μg/ml). One group of mice was euthanized at 4 and another at 24 hours following stimulation, the urinary bladders were removed from all groups (n = 8) for RNA extraction.

#### Sample preparation for cDNA expression arrays

We used the same technology as we described before [6,20,21,39,40]. Briefly, eight bladders from each group were homogenized together in Ultraspec RNA solution (Biotecx Laboratories Inc. Houston, TX) for isolation and purification of total RNA. Mouse bladders were pooled to ensure enough RNA for gene array analysis. The justification for this approach is that there is not enough RNA in a single mouse bladder for performing cDNA array experiments and the step of purification reduces the amount of total RNA. RNA was DNase-treated according to manufacturer's instructions (Clontech Laboratories, Palo Alto, CA), and the quality of 10 μg was evaluated by denaturing formaldehyde/agarose gel electrophoresis.

#### Mouse cDNA expression arrays

cDNA probes were prepared from DNase-treated RNAs obtained from each of the experimental groups. Five μg of DNase-treated RNA was reverse-transcribed to cDNA and labeled with [α-^33^P]dATP, according to the manufacturer's protocol (Clontech, Palo Alto, CA). The radioactively labeled complex cDNA probes were hybridized overnight to Atlas™ mouse plastic 5 K arrays (Clontech, Palo Alto, CA) using ExpressHyb™ hybridization solution with continuous agitation at 68 °C. After two high-stringency washes, the hybridized membranes were exposed (at room temperature) to a ST Cyclone phosphor screen overnight. Spots on the arrays were quantified by BD AtlasImage™ 2.7 software (Clontech, Palo Alto, CA).

#### PAR1-dependent genes. Both the 4 hour and 24 hour time points after inflammation were used to define PAR1-dependent genes

Data was normalized by a robust linear regression analysis using only genes expressed above background, as described [7,20,39,40], and the ratio of gene-expression between LPS- and SP-, and saline-challenge was obtained at 4 and 24 hours post-challenge. PAR1^-/- ^dependent genes were selected according to the following criterion: **A. **In tissues isolated from WT mice, the expression of particular gene should be up-regulated (ratio between LPS- or SP- and saline-treated > 3.0) in at least one of the time points (4 and 24 hours post-challenge); **B. **in tissues isolated from PAR1^-/- ^mice, the expression of same gene should not be altered in response to LPS or SP in any of the time points.

### Database submission of microarray data

The microarray data have been deposited in the Gene Expression Omnibus (GEO) database [92]. The samples can be retrieved with GEO accession numbers: GSM144550, GSM144551, GSM144552, GSM144553, GSM144554, GSM144555, GSM144556, GSM144557, GSM144558, GSM144559 that are included in a series (accession number GSE6286).

### Development of the observed interactome of PAR1-specific genes

Ingenuity Pathways Analysis [IPA], (Ingenuity Systems, Mountain View, CA) is a robust and expertly curated database containing up-to-date information on over 20,000 mammalian genes and proteins, 1.4 million biological interactions, and one hundred canonical pathways incorporating over 6,000 discreet gene concepts. This information is integrated with other relevant databases such as EntrezGene and Gene Ontology [29]. IPA computes a score for each network according to the fit of the set of supplied focus genes (here, PAR1-dependent genes). These scores, derived from *p *values, indicate the likelihood of focus genes to belong to a network versus those obtained by chance. A score > 2 indicates a ≤ 99% confidence that a focus gene network was not generated by chance alone [93]. The experimental datasets of PAR1-dependent genes was used to query the IPA and to compose a set of interactive networks taking into consideration canonical pathways, the relevant biological interactions, and the cellular and disease processes.

### Target validation by Q-PCR of Chromatin Immunoprecipitation (CHIP)-based assays

Target validation was sought for 19 of the genes identified from the microarray data as being PAR1-dependent. Female C57BL/6J mice (n = 20 per group) were anesthetized (ketamine 200 mg/kg and xylazine 2.5 mg/kg, i.p.), then transurethrally catheterized (24 Ga.; 3/4 in; Angiocath, Becton Dickson, Sandy, Utah), and the urine was drained by applying slight digital pressure to the lower abdomen. The urinary bladders were instilled with 200 μl of one of the following substances: control inactive peptide (LRGILS [94]) or PAR-activating peptides (PAR1-AP = SFFLRN [94]; PAR2-AP = SLIGRL [94]). Substances were infused at a slow rate to avoid trauma and vesicoureteral reflux (18). To ensure consistent contact of substances with the bladder, infusion was repeated twice within a 30-min interval, and a 1-ml tuberculin syringe was maintained on the catheter end to retain the intravesical solution for 1 hour. After that the catheter was removed and mice were allowed to void normally. Twenty-four hours after instillation, mice were euthanized with pentobarbital (200 mg/kg, i.p.) and bladders removed rapidly and frozen.

Frozen bladders were shipped to Genpathway [95] for querying the chromatin for transcription of specific genes (Genpathway's TranscriptionPath Query assay) [96]. Bladders were exposed briefly to formaldehyde for cross-linking of the proteins and DNA together, followed by sonication to fragment the DNA into pieces of approximately 300–500 bp. An antibody against RNA polymerase II (Abcam) was then used to precipitate the DNA transcriptome. The Ab-protein-DNA complexes were purified using beads coupled to protein A. The DNA was isolated from the complexes using a combination of heat to reverse cross-linking, RNase and proteases, and then purified using phenol extraction and EtOH precipitation. The final CHIP DNAs were then used as templates for Q-PCR reactions using primer pairs specific for each gene of interest. Q-PCR was carried out using Taq polymerase (iQ SYBR Green Supermix, Bio-Rad). Primer pairs were designed using Primer 3 [97]. Details of the primer sequences and the Genebank accession numbers are given in additional material 2 (Table 2). The designed primers shared 100% homology with the target sequence but no significant homology with other sequences.

Q-PCRs were run in triplicate and the averaged Ct values were transferred into copy numbers of DNA using a standard curve of genomic DNA with known copy numbers. The resulting transcription values for each gene were also normalized for primer pair amplification efficiency using the Q-PCR values obtained with Input DNA (unprecipitated genomic DNA). Results are presented as Transcription Events Detected Per 1000 Cells for each gene tested.

### Tissues for Western Blotting

Twenty-four hours post-instillation, bladders were rapidly removed and placed in ice cold PBS with protease inhibitors (Complete Protease Inhibitor, Roche, Indianapolis, IN) on ice where the mucosa was dissected away from the detrusor smooth muscle, as described [32]. Tissues were flash frozen and stored at -80 C until processing. Tissues were pulverized in a spring-loaded tissue pulverizer (Bio-Pulverizer, Biospec Products, Bartlesville, OK) and chilled with liquid nitrogen. Cytosolic extracts were prepared using the Pierce NE-PER Kit that enables stepwise separation and preparation of cytoplasmic and nuclear extracts from bladder tissue. Addition of the first two reagents (Pierce's proprietary information) to the pulverized tissue causes disruption of cell membranes and release of cytoplasmic contents. After recovering the intact nuclei from the cytoplasmic extract by centrifugation at 16,000 × g for 5 minutes, the nuclei are lysed with a third reagent (Pierce's proprietary information) to yield the nuclear extract. Extracts obtained with this product generally have less than 10% contamination between nuclear and cytoplasmic fractions–sufficient purity for most experiments involving nuclear extracts. A western blot was prepared using the nuclear and cytosolic extracts and probed for the nuclear proteins histone H3 and lamin A/C. No nuclear contamination was shown in the cytosolic fractions (data not shown). Protein concentrations were determined with a Micro BCA Kit (Pierce, Rockford, IL) per manufacturer's instructions.

### Western Blot

Immunoblot analyses were performed using 15 μg cytosolic extract loaded onto a 10% tris-glycine SDS gel and run at 125 V for 1.5 hours in tris-glycine SDS running buffer (BioRad, Hercules, CA). The proteins were then transferred to a 0.45 μm nitrocellulose membrane in 1/2 × tris-glycine SDS running buffer with 20% methanol using a BioRad Mini-TransBlot Cell. After blocking in 2% BSA, blots were incubated with rabbit anti-iPLA_2 _(Cayman Chemical, Ann Arbor, MI) at 1:500 overnight at 4°C. An HRP-conjugated anti-rabbit secondary antibody was used for detection at 1:10,000 (Santa Cruz Biotechnology, Santa Cruz, CA) and an enhanced chemiluminescent detection kit (Chemiglow West, Alpha Innotech, San Leandro, CA) was used to visualize. Images were taken using the FluorChem HD digital darkroom (Alpha Innotech, San Leandro, CA) and quantified using ImageJ [98].

### Materials

PAR-AP – PAR1-, PAR2-, and PAR4-AP were synthesized at Molecular Biology Resource Facility, William K. Warren Medical Research Institute, OUHSC, as carboxyl-terminal amides, purified by high-pressure liquid chromatography, and characterized by mass spectroscopy. Peptide solutions were made fresh from powder for most experiments. PAR3-AP was not used in this research because of lack of specificity.

## Competing interests

The author(s) declare that they have no competing interests.

## Authors' contributions

All authors read and approved the final manuscript. **RS **conceived of the study and drafted the manuscript, **MRD **participated in the design and reviewed the morphological results, **PAG **participated in the experimental design and provided PAR1^-/- ^and PAR2^-/- ^mice, **CKD **participated in design and proper use of PAR-APS, **ID **performed the statistical analysis of microarray results, **MI **participated in its design and helped to draft the manuscript, **REH **participated in its design and helped to draft the manuscript, **CS **performed the western blotting analysis and helped **MRS **with animal experiments, and **MRS **participated in its design, carried out the animal experiments, removed the tissues, and performed gene array experiments.

## Supplementary Material

Additional file 1 PAR1-Dependent Transcripts. Table 1 Click here for file

Additional file 2 Primers for Q-PCR. Table 2 Click here for file
