# Supplementary material for: Regulatory network of inflammation downstream of proteinase-activated receptors
Source: BMC Physiol. 2007 Mar 30;7:3. doi: 10.1186/1472-6793-7-3 (PMC1853107; doi:10.1186/1472-6793-7-3)
Supplement: Additional file 1 — PAR1-Dependent Transcripts. Table 1 [file 1472-6793-7-3-S1.pdf]

TABLE 1A PAR1- DEPENDENT TRASNCRIPTS

SYMBOL	*GENENAME*	*LOCUS (Entrez Gene ID)*	*MOUSE REF*
ACTB	actin, beta, cytoplasmic	11461	NM_007393
ACTG1	actin, gamma, cytoplasmic	11465	NM_009609
ACVR2B / ActR-IIB	activin receptor IIB	11481	NM_007397
ADAM3	a disintegrin and metalloprotease domain 3 (cyritestin)	11497	NM_009619
AICDA	activation-induced cytidine deaminase	11628	NM_009645
AKT2	thymoma viral proto-oncogene 2	11652	NM_007434
ALPL/AKP2	alkaline phosphatase 2, liver	11647	NM_007431
ARF6	ADP-ribosylation factor 6	11845	NM_007481
ASTN2	astrotactin 2	56079	NM_019514
ATBF1	AT motif binding factor 1	11906	NM_007496
B2M	beta-2 microglobulin	12010	NM_009735
BANF1	barrier to autointegration factor 1	23825	NM_011793
BTG3	B-cell translocation gene 3	12228	NM_009770
CCL7	chemokine (C-C motif) ligand 7	20306	NM_013654
CD200 / MOX2	antigen identified by monoclonal antibody MRC OX-2	17470	NM_010818
CD63	Cd63 antigen	12512	NM_007653
CEBPD	CCAAT/enhancer binding protein (C/EBP), delta	12609	NM_007679
CFD / ADN	complement factor D/ adipsin	11537	NM_013459
CFL1	cofilin 1, non-muscle	12631	NM_007687
CRSP2	cofactor required for Sp1 transcriptional activation, subunit 2, 150kDa	26896	NM_012005
CSTB	cystatin B (stefin B)	13014	NM_007793
CUL3	cullin 3	26554	NM_016716
DCTN1	dynactin 1/p150 GLUED	13191	NM_007835
DDOST	dolichyl-di-phosphooligosaccharide-protein glycotransferase	13200	NM_007838
DUSP1	dual specificity phosphatase 1	19252	NM_013642
DVL3	dishevelled 3, dsh homolog (Drosophila)	13544	NM_007889
ELK1	ELK1, member of ETS oncogene family	13712	NM_007922
FKBP1A	FK506 binding protein 1a	14225	NM_008019
FMNL1	formin-like	57778	NM_019679
FTH1	ferritin heavy chain	14319	NM_010239
FXYD3	FXYD domain-containing ion transport regulator 3	17178	NM_008557
GAPDH	glyceraldehyde-3-phosphate dehydrogenase, spermatogenic	14447	NM_008085
GFPT2	glutamine fructose-6-phosphate transaminase 2	14584	NM_013529
GUCA2B	guanylate cyclase activator 2b (retina)	14916	NM_008191
HMGA1/ HMG-I(Y)	high mobility group AT-hook 1	15361	NM_016660
HSPB1 / HSP 27	heat shock protein 27, protein 1	15507	NM_013560
INA	internexin neuronal intermediate filament protein, alpha-internexin	226180	NM_146100
JUND	Jun proto-oncogene related gene d1	16478	NM_010592
KRT1 / KRTHA2A	keratin complex 1, acidic, gene 1	16660	NM_010659
MAN2B2	mannosidase 2, alpha B2	17160	NM_008550
MARCKSL1 / MLP	MARCKS-like protein	17357	NM_010807
MID2	midline 2	23947	NM_011845
MMP2	matrix metalloproteinase 2	17390	NM_008610
MYADM	myeloid-associated differentiation marker	50918	NM_016969
MYL6B/ myo1a	cDNA sequence BC037527 myosin light chain 1 slow a	216459	NM_172259
MYO5A	myosin Va	17918	NM_010864
NEU2	neuraminidase 2	23956	NM_015750
NFKBIA	nuclear factor of kappa light chain gene enhancer in B-cells inhibitor, alpha	18035	NM_010907
NKX2-6	NK2 transcription factor related, locus 6 (Drosophila)	18092	NM_010920
NR1I3	nuclear receptor subfamily 1, group I, member 3	12355	NM_009803
PAX1	paired box gene 1	18503	NM_008780
PHLDA1/ TDAG51	pleckstrin homology-like domain, family A, member 1	21664	NM_009344
PLA2G1B	phospholipase A2, group IB, pancreas	18778	NM_011107
PLAUR / u-PAR	urokinase plasminogen activator receptor	18793	NM_011113
PPIA	peptidylprolyl isomerase A	268373	NM_008907
PRG1 / serglycin	proteoglycan, secretory granule	19073	NM_011157
PTPRCAP / CD45	protein tyrosine phosphatase, receptor type, C polypeptide-associated protein	19265	NM_016933
PSG19	pregnancy specific glycoprotein 19	26439	NM_011964
PTP4A2	protein tyrosine phosphatase 4a2	19244	NM_008974
PTPN1	protein tyrosine phosphatase, non-receptor type 1	19246	NM_011201
PXMP3	peroxisomal membrane protein 3	19302	NM_008994
RAD9A	RAD9 homolog (S. pombe)	19367	NM_011237
RPS29	ribosomal protein S29	20090	NM_009093
S100A10	S100 calcium binding protein A10 (calpactin)	20194	NM_009112
SIM2	single-minded 2	20465	NM_011377
SKP1A	S-phase kinase-associated protein 1A	21402	NM_011543
SNRPC	U1 small nuclear ribonucleoprotein 1C	20630	NM_011432
SYNGR1	synaptogyrin 1	20972	NM_009303
SM22a / TAGLN	transgelin	21345	NM_011526
TNFAIP3/A20	tumor necrosis factor, alpha-induced protein 3	21929	NM_009397
UBE2H / UbcH2	ubiquitin-conjugating enzyme E2H	22214	NM_009459
UBR1	ubiquitin protein ligase E3 component n-recognin 1	22222	NM_009461
UPK2	uroplakin 2	22269	NM_009476
WISP2	WNT1 inducible signaling pathway protein 2	22403	NM_016873
WNT6	wingless-related MMTV integration site 6	22420	NM_009526

TABLE 1B ANNOTATION OF PAR1- DEPENDENT TRASNCRIPTS

SYMBOL	*COMPONENT*	*Family*	*Molecular Function*	*Biological Process*
ACTB	Cytoplasm		Motor activity	
ACTG1	Cytoplasm		Structural molecule activity/ motor activity	Cytoskeleton/ Actin filament/ Sarcomere organization
ACVR2B / ActR-IIB	Plasma Membrane	kinase		
ADAM3	Plasma Membrane	peptidase		
AICDA	Cytoplasm	enzyme	Cytidine deaminase activity	mRNA processing
AKT2	Cytoplasm	kinase	nucleotide binding	protein amino acid phosphorylation/ JNK cascade
ALPL/AKP2	Plasma Membrane	phosphatase		
ARF6	Plasma Membrane	transporter		
ASTN2	Unknown	other		
ATBF1	Nucleus	transcription regulator	Transcription factor activity/DNA binding	Regulation of transcription
B2M	Plasma Membrane	transmembrane receptor		
BANF1	Nucleus		DNA binding	DNA integration/ Provirus integration
BTG3	Nucleus		Protein binding	Negative regulation of cell cycle
CCL7	Extracellular Space	cytokine	chemokine activity/ heparin binding	Inflammation/ chemotaxis
CD200 / MOX2	Plasma Membrane			
CD63	Plasma Membrane			
CEBPD	Nucleus	transcription regulator	Protein homodimerization / DNA binding	Inflammation/ regulation of transcription
CFD / ADN	Extracellular Space	peptidase	Endopeptidase/ Trypsin activity/ Complement Factor	Immune response/ complement activation
CFL1	Nucleus		Actin binding	Establishment of cell polarity/ neurocrest migration
CRSP2	Nucleus	transcription regulator	Transcription coavtivator	Positive regulaion of transcription
CSTB	Cytoplasm		Endopeptidase inhibitor / Cysteine protease inhibitor activity	adult locomotory behavior
CUL3	Nucleus			Cell cycle/ubiquitin
DCTN1	Cytoplasm		Motor activity/ Protein binding	
DDOST	Cytoplasm	enzyme	Transfease activity	N-linked glycosylation
DUSP1	Nucleus	phosphatase	MAP kinase activity	Cell cycle/protein and amino acid dephosphorylation
DVL3	Cytoplasm		Signal transducer activity/ Protein bining	Wnt receptor signalling/frizzle signaling/development
ELK1	Nucleus	transcription regulator	Transcription factor activity/DNA binding	Cell physiological process/ cell cycle/ transcription
FKBP1A	Cytoplasm	enzyme	ATP binding/FK506 binding	Protein folding/transport
FMNL1	Cytoplasm		Profilin binding	Cellular physiology/ Substrate-bound cell migration
FTH1	Cytoplasm	enzyme	Ferric iron binding	Iron ion homeostasis
FXYD3	Plasma Membrane	ion channel		
GAPDH	Cytoplasm	enzyme		
GFPT2	Cytoplasm	enzyme		
GUCA2B	Extracellular Space		enzyme activator activity	cGMP biosynthesis/ fluid secretion
HMGA1/ HMG-I(Y)	Nucleus	transcription regulator	DNA binding	Chromosome organization/ spermatogenesis
HSPB1 / HSP 27	Cytoplasm			Protein folding/ regulation of translation initiation
INA	Cytoplasm		Structural molecule activity	Cytoskeleton/ Cell differentiation
JUND	Nucleus	transcription regulator	Trancription factor/ DNA binding activity	Cell physiology process/ Regulation of transcription
KRT1 / KRTHA2A	Cytoplasm			
MAN2B2	Cytoplasm	enzyme	hydrolase activity	carbohydrate metabolism
MARCKSL1 / MLP	Cytoplasm		Calmodulin binding	
MID2	Cytoplasm		Ubiquitin-protein ligase activity	Protein ubiquitination
MMP2	Extracellular Space	peptidase	Metalloendopeptidase/gelatinase A activity	Proteolysis/ Collagen catabolism
MYADM	Nucleus			
MYL6B	Cytoplasm	peptidase		
MYO5A	Cytoplasm	enzyme	actin binding/ calmodulin binding/ ATP binding	Actin filament-base movement/ transport
NEU2	Cytoplasm	enzyme	hydrolase activity	carbohydrate metabolism
NFKBIA	Cytoplasm		Protein binding	Regulation of cell proliferation
NKX2-6	Nucleus	transcription regulator	Transcription factor activity/DNA binding	Regulation of transcription/ Development
NR1I3	Nucleus	ligand-dependent nuclear receptor	Nuclear Receptor/ Steroid hormone receptor activity	Negative regulation of transcription
PAX1	Nucleus	transcription regulator	Transcription factor activity/DNA binding	Transcription/ Organogenesis
PHLDA1/ TDAG51	Cytoplasm			FASL byosynthesis
PLA2G1B	Extracellular Space	enzyme	Phospholipase A2 activity	phospholipid catabolism/ stress/ cell proliferation
PLAUR / u-PAR	Plasma Membrane	transmembrane receptor		
PPIA	Cytoplasm	enzyme	Isomerase activity/peptidyl prolyl cis-trans isomerase	Protein folding
PRG1 / serglycin	Extracellular Space			
PTPRCAP / CD45	Plasma Membrane	phosphatase		
PSG19	Extracellular Space			Pregnancy/ cancer?
PTP4A2	Plasma Membrane	phosphatase		
PTPN1	Cytoplasm	phosphatase	Phosphoprotein/ Hydrolase activity	protein amino acid dephosphorylation
PXMP3	Cytoplasm		ubiquitin-protein ligase activity/ zinc ion binding/	Neurogenesis/ peroxisome organization
RAD9A	Nucleus			DNA damage/ Response to radiation
RPS29	Cytoplasm		zinc ion binding/RNA binding	Protein synthesis/ Ribosome biogenesis
S100A10	Cytoplasm		calcium ion binding	
SIM2	Nucleus	transcription regulator	Signal transducer activity	Cell differentiation/ neurogenesis
SKP1A	Nucleus	transcription regulator		
SNRPC	Nucleus			
SYNGR1	Plasma Membrane	transporter		
SM22a / TAGLN	Cytoplasm		Muscle development	
TNFAIP3/A20	Nucleus		zinc ion activity/DNA binding	Ubiquitin cycle/ apoptosis
UBE2H / Ubch2	Cytoplasm	enzyme	ubiquitin-protein ligase activity	Ubiquitin cycle/ Protein modification
UBR1	Cytoplasm	enzyme	ubiquitin-protein ligase activity	Ubiquitin cycle
UPK2	Plasma Membrane			
WISP2	Extracellular Space	growth factor	Phospholipase A2 activity	phospholipid catabolism
WNT6	Extracellular Space		singnal transduction/ protein binding/ receptor binding	Wnt receptor pathway/ frizzled-2 signalling
