# Supplementary material for: Regulatory network of inflammation downstream of proteinase-activated receptors
Source: BMC Physiol. 2007 Mar 30;7:3. doi: 10.1186/1472-6793-7-3 (PMC1853107; doi:10.1186/1472-6793-7-3)
Supplement: Additional file 2 — Primers for Q-PCR. Table 2 [file 1472-6793-7-3-S2.pdf]

TABLE 2 PRIMERS FOR Q-PCR

SYMBOL	*MOUSE REF*	*FORWARD PRIMER*	*REVERSE PRIMER*
Actb	NM_007393	tgataagtggccttggagtg	ctcagggcaggtgaaactgt
Akt2	NM_007434	acagttggcctcttggtgag	ccctgcttagggaccacag
Arf6	NM_007481	acgccatcatcctcatcttc	agggctgcacataccagttc
Ccl7	NM_013654	aactgaagcccaccactctc	atgacccaggaatggatgag
Cd63	NM_007653	gcacccacttctctgctctt	gccatcccactcacttcttt
Dusp1	NM_013642	gtcgccactcctcctgtg	gcggtcaagtcattgttgtg
Fkbp1a	NM_008019	agcaacaaaccaggcacaag	aggacttttccaacggtctc
Mmp2	NM_008610	tgcctccaagcaggtaagag	accatgtgtcaatccctgtg
Nfkbia	NM_010907	tggcctccaaacacacagt	cctggtgcccttgttctatt
Phlda1	NM_009344	agggcggggagagaagag	ggaggatggcactgaacaag
Pla2g1b	NM_011107	acctcctgtcctaccgactg	ttccacctgtctccatctctg
Plaur	NM_011113	cccaacaaccgatagcaaac	atctggctggctacctgaac
S100a10	NM_009112	aaataatggatgtttgtgttcactg	agcaatcccaaatgtctgttc
Tnfaip3	NM_009397	ggtgcccgactggtattaag	ctgtgggtcaatgctgagac
Ube2h	NM_009459	aagaaaggcaaagatgcactg	ttttcactcccaaactacattaaac
Upk2	NM_009476	ccgagtacagaaggggacat	tcggagcttagggaagtgtg
